# Acute Liver Injury of Unclear Etiology: A Case of Alcohol-Associated Steatohepatitis

**DOI:** 10.7759/cureus.89223

**Published:** 2025-08-01

**Authors:** Zohayr A Khan, Abigail L Ellington, Wakeem L Abraham, Xianyong Gui, William C Lippert

**Affiliations:** 1 Internal Medicine, Wake Forest University School of Medicine, Winston-Salem, USA; 2 Pathology, Wake Forest University School of Medicine, Winston-Salem, USA

**Keywords:** acute liver injury, alcohol-associated steatohepatitis, liver biopsy, liver inflammation, steatohepatitis, wilson’s disease

## Abstract

A 37-year-old female was hospitalized for acute liver injury, presenting a diagnostic challenge that ultimately led to a diagnosis of alcohol-associated steatohepatitis (ASH). Steatohepatitis is a state of liver inflammation with fat accumulation and has several potential etiologies, including metabolic dysfunction-associated, alcohol-associated, drug-induced, autoimmune, and viral causes. A definitive diagnosis often requires a thorough clinical history, laboratory and imaging studies, and, in some cases, a liver biopsy. Our patient, with a history of gastroesophageal reflux disease, alcohol-use disorder, obesity, and systemic lupus erythematosus, presented with a one-week history of abdominal pain, jaundice, conjunctival icterus, and hepatomegaly without altered mental status. Laboratory results showed a total bilirubin of 12.5 mg/dL, aspartate aminotransferase of 237 U/L, alanine aminotransferase of 129 U/L, alkaline phosphatase of 424 U/L, and an international normalized ratio of 1.2. Right upper quadrant ultrasound revealed no biliary obstruction. Viral and autoimmune panels, as well as immunoglobulins, were unremarkable. Ceruloplasmin was low (0.09 g/L), 24-hour urinary copper was elevated (40 µg/24 hour), and slit-lamp examination was negative for Kayser-Fleischer rings. Liver biopsy revealed severe steatosis with prominent Mallory-Denk bodies, bridging fibrosis, and portal tract inflammatory infiltrates. These histologic features, together with a positive phosphatidylethanol test, led to the diagnosis of ASH. This case highlights an interesting diagnostic challenge of acute liver injury. Although ceruloplasmin was low and 24-hour urinary copper was elevated, these levels did not meet the diagnostic criteria for Wilson’s disease. Liver biopsy suggested ASH, despite the patient reporting cessation of alcohol use. In cases of acute liver injury where history, labs, and imaging studies are inconclusive, liver biopsy is crucial for diagnosis. Treatment for ASH includes abstinence from alcohol and corticosteroids.

## Introduction

Alcohol-associated steatohepatitis (ASH) is a condition of acute liver inflammation and fat accumulation precipitated by overconsumption of alcohol (>40 g or >50-60 g daily over six months for women and men, respectively), with typical laboratory findings of elevated total bilirubin and liver enzymes [1]. At a molecular level, ASH is caused by alcohol consumption increasing gut permeability, while also causing direct damage to hepatocytes, which then release pro-inflammatory molecules [1]. Diagnosis is made based on clinical history, laboratory and imaging findings, and liver biopsy, with the differential typically including viral, autoimmune, and metabolic dysfunction-associated liver disease. In patients who present with acute liver injury of uncertain etiology, a liver biopsy can help make a definitive diagnosis, particularly in the setting of ASH. Treatment for ASH, beyond abstinence from alcohol, consists of corticosteroids and occasionally N-acetylcysteine. We present the case of a patient with a history of alcohol-use disorder who presented to the hospital with acute liver injury, concerning for alcohol-related liver disease. However, laboratory findings also raised suspicion for Wilson’s disease. A comprehensive hepatological workup, along with a liver biopsy, ultimately confirmed the diagnosis of ASH. Given how likely clinicians are to encounter patients who consume alcohol, they need to recognize this clinical entity and be able to differentiate it from other causes of liver injury.

## Case presentation

A 37-year-old female with a history of gastroesophageal reflux disease, alcohol-use disorder, systemic lupus erythematosus (SLE), and obesity, but no other cardiometabolic risk factors, presented to an outside emergency department (ED) with a one-week history of right upper quadrant (RUQ) pain and abdominal bloating. At the outside ED, the patient was found to have elevated liver enzymes and bilirubin, with CT of the abdomen and pelvis showing no acute intra-abdominal pathology, but moderate hepatomegaly and marked hepatic steatosis. Based on these results, the patient required further gastroenterology evaluation and was transferred to our academic hospital, where an extensive workup commenced. Upon further history, a family member had noticed yellowing of the patient’s eyes. Of note, the patient reported recently discontinuing alcohol use through a gradual self-taper and reported no alcohol consumption for at least 10 days before presentation. Previously, she consumed up to 12 drinks daily for the preceding three years. On physical examination at our hospital, the patient had a body mass index (BMI) of 33 kg/m^2^, jaundice, conjunctival icterus, and mild, tender hepatomegaly, but no altered mental status. Initial laboratory findings at our hospital (Table 1) included a total bilirubin of 12.5 mg/dL (normal = 0.3-1.0 mg/dL), a direct bilirubin of 7.7 mg/dL (normal = 0.0-0.2 mg/dL), aspartate aminotransferase (AST) of 237 U/L (normal = 13-39 U/L), alanine aminotransferase (ALT) of 129 U/L (normal = 7-52 U/L), alkaline phosphatase (ALP) of 424 U/L (normal = 34-104 U/L), international normalized ratio (INR) of 1.2 (normal = 0.8-1.1), and albumin of 3.0 g/dL (normal = 3.5-5.7 g/dL), suggestive of a cholestatic pattern of liver injury. Based on these initial laboratory findings, the patient’s Model for End-Stage Liver Disease (MELD) score was 23. Viral hepatitis and autoimmune panels (rheumatoid factor, dsDNA, anti-Smith, AMA) were negative, but ANA and anti-RNP antibodies were positive at 1:320 and 1.9, respectively. Immunoglobulins were within the normal range. RUQ ultrasound revealed hepatomegaly and steatosis but no biliary obstruction.

**Table 1:** Laboratory findings. WBC = white blood cells; RBC = red blood cells; AST = aspartate aminotransferase; ALT = alanine aminotransferase; ALP = alkaline phosphatase; INR = international normalized ratio; IgM = immunoglobulin M; dsDNA = double-stranded DNA; IgG = immunoglobulin G; ANA = anti-nuclear antibody; AMA = anti-mitochondrial antibody; RNP = ribonucleoprotein; IgA = immunoglobulin A; PEth = phosphatidylethanol

Test	Result	Reference range	Unit
Complete blood count			
WBC	5.20	4.40–11.0	10^3^/µL
RBC	4.36	4.10–5.10	10^6^/µL
Hemoglobin	13.7	12.3–15.3	g/dL
Mean corpuscular volume	89.9	80.0–96.0	fL
Platelets	64	150–450	10^3^/µL
Comprehensive metabolic panel and liver function tests			
Sodium	129	136–145	mmol/L
Potassium	3.5	3.5–5.1	mmol/L
Chloride	93	98–107	mmol/L
Bicarbonate	24	21–31	mmol/L
Anion gap	12	6–14	mmol/L
Glucose	111	70–99	mg/dL
Blood urea nitrogen	2	7–25	mg/dL
Creatinine	0.92	0.60–1.20	mg/dL
Calcium	8.1	8.6–10.3	mg/dL
Albumin	3.0	3.5–5.7	g/dL
Total protein	5.5	6.4–8.9	g/dL
Total bilirubin	12.5	0.3–1.0	mg/dL
Direct bilirubin	7.7	0.0–0.2	mg/dL
AST	237	13–39	U/L
ALT	129	7–52	U/L
ALP	424	34–104	U/L
INR	1.2	0.8–1.1	
Acute hepatitis panel			
Hepatitis B surface antigen	Non-reactive	-	-
Hepatitis B core antibody, IgM	Non-reactive	-	-
Hepatitis A antibody, IgM	Non-reactive	-	-
Hepatitis C virus antibody	Non-reactive	-	-
Autoimmune panel			
Rheumatoid factor	<7.0	<12.5	IU/mL
dsDNA antibody, IgG	<10	0–99	IU/mL
Smith antibodies, IgG	<0.2	<1.0	U
ANA	1:320	<1:80	-
AMA	<20.0	0.0–20.0	U
RNP antibodies, IgG	1.9	<1.0	U
Immunoglobulins			
IgA	383	66–433	mg/dL
IgG	901	635–1,741	mg/dL
IgM	73	45–281	mg/dL
Copper metabolism			
Ceruloplasmin	0.09	0.2–0.6	g/L
24-hour urinary copper	40	3–35	µg
Alcohol			
PEth	>2,000	<20	ng/mL

**Figure 1:**
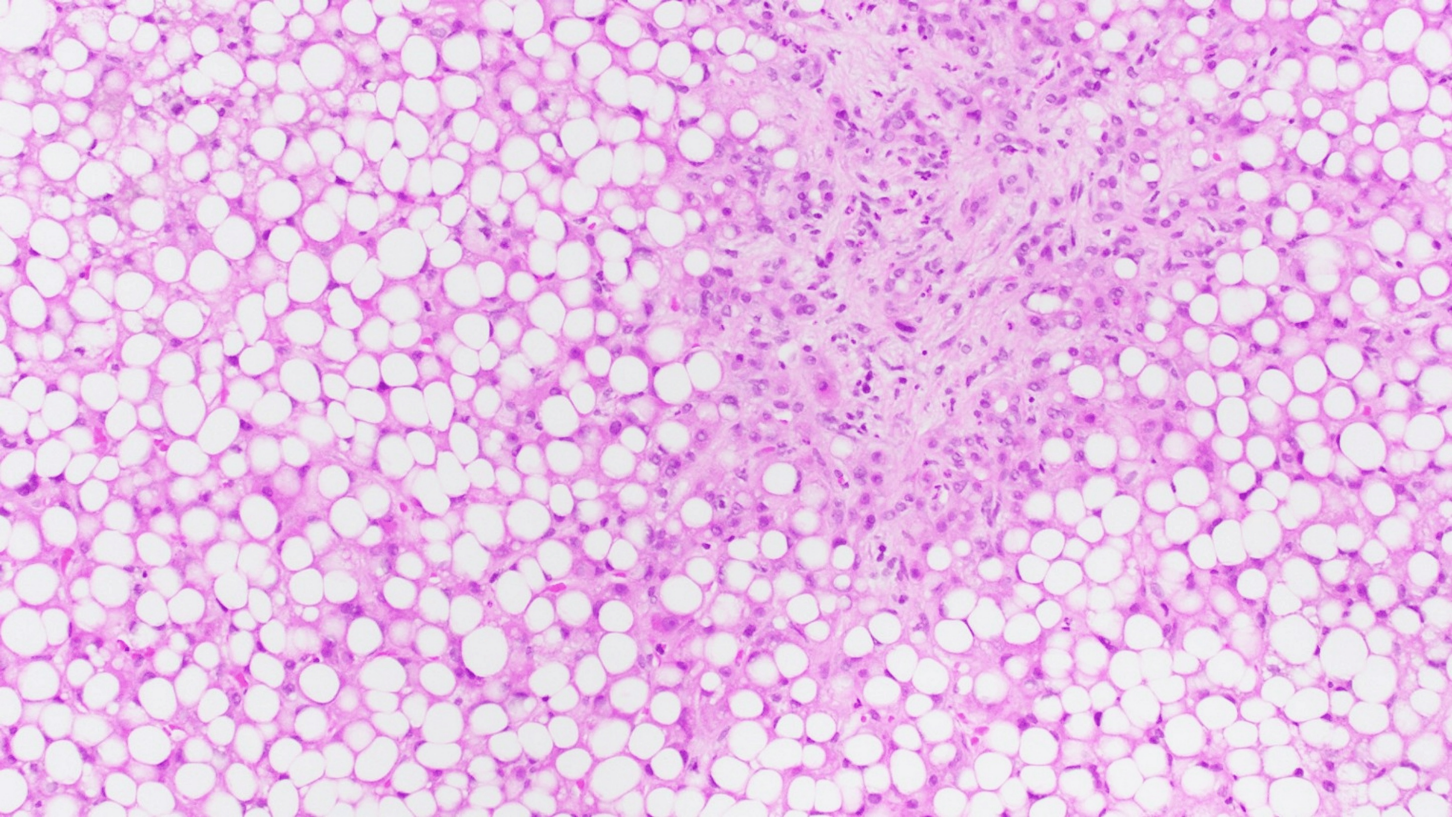
Liver biopsy demonstrating severe macrovascular steatosis with neutrophil-rich portal inflammation and fibrosis (10×).

**Figure 2:**
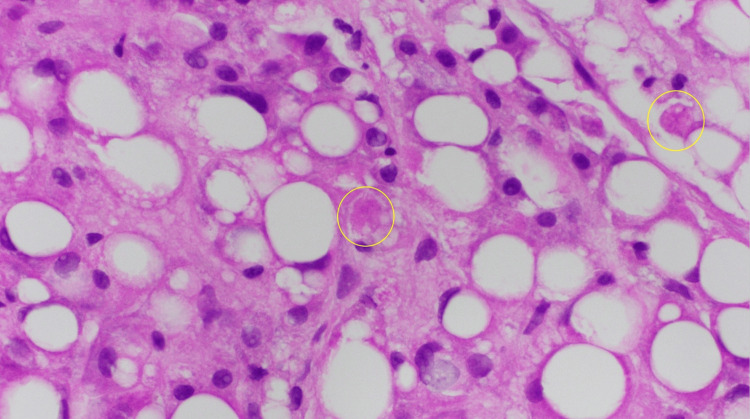
Frequent Mallory-Denk bodies (circled) on liver biopsy (20×).

Wilson’s disease was considered due to a low ceruloplasmin level at 0.09 g/L (normal = 0.2-0.6 g/L) and an elevated 24-hour urinary copper of 40 µg (normal = 3-35 µg). However, a slit-lamp examination was negative for Kayser-Fleischer rings, which are present in nearly all patients with neurologic Wilson’s disease and in approximately 50% with hepatic Wilson’s disease [2]. Given the unclear etiology of liver injury in the setting of abnormal Wilson’s disease markers and prior alcohol use, the decision was made to conduct a liver biopsy. It revealed severe macrovascular steatosis with neutrophil-rich portal inflammation and fibrosis (Figure 1) and Mallory-Denk bodies (Figure 2), which are relatively more common in ASH, with one study reporting a 65% prevalence [3]. A phosphatidylethanol (PEth) test, a long-term biomarker for large alcohol consumption (sensitivity and specificity of ~95-99% and 100%, respectively) [4], was ordered and returned positive at >2000 ng/mL (normal <20 ng/mL), further supporting ASH. Given worsening liver function (Maddrey’s Discriminant Function of 44, suggesting poor prognosis), oral prednisolone dosed at 40 mg daily was started, with subsequent improvement in AST, ALT, and ALP. Given the liver biopsy findings, positive PEth test, and response to corticosteroids, the patient was diagnosed with ASH and later discharged to continue 40 mg prednisolone daily for a total of 28 days. She was seen in the outpatient clinic after completion of her 28-day course and had near normalization of her bilirubin, AST, ALT, and ALP levels.

## Discussion

This case highlights the diagnostic complexity of acute liver injury of an initial unknown origin. Acute liver injury is a state of liver dysfunction characterized by elevated transaminases (i.e., ALT, AST) and impaired synthetic liver function without hepatic encephalopathy (HE); in contrast, the presence of HE indicates acute liver failure [5]. The initial differential for our patient was broad, including ASH, metabolic dysfunction-associated steatohepatitis, autoimmune etiologies, viral hepatitis, drug or toxin-induced hepatitis, and Wilson’s disease. Viral and drug-induced hepatitis were ruled out based on laboratory findings and history. Autoimmune hepatitis was of concern in the setting of the patient’s prior history of SLE and prior cases of patients with SLE developing autoimmune hepatitis [6]. However, the autoimmune panel was negative. ASH, given the patient’s history of alcohol-use disorder, remained high on the differential. Wilson’s disease was a concern due to the patient’s low ceruloplasmin of 0.09 g/L being within the diagnostic range for Wilson’s disease (<0.20 g/L). While the 24-hour urinary copper was elevated (40 µg), it was not as high as usually seen in Wilson’s disease (>100 µg) [7], and the elevation was thought to be due to the patient’s underlying liver injury, given the lack of ability to metabolize copper. PEth, which detects alcohol consumption for up to four weeks prior, further pointed toward a diagnosis of ASH. However, our patient had reportedly recently discontinued alcohol consumption; therefore, a positive PEth was not surprising. Ultimately, given the diagnostic challenge presented and inconclusive initial workup, the patient underwent liver biopsy, which helped confirm the diagnosis of ASH. The liver biopsy’s finding of neutrophil-rich inflammation and frequent Mallory-Denk bodies is a feature favoring ASH [8]. Autoimmune hepatitis would have shown notable plasma cell-predominant portal inflammation and interface hepatitis [9], while Wilson’s disease may show copper deposition within hepatocytes, using a special rhodamine stain [10]. While metabolic dysfunction-associated steatohepatitis and metabolic dysfunction-associated fatty liver disease were of concern given the patient’s BMI (33 kg/m^2^) and the histological findings on liver biopsy, the patient’s history of alcohol use, positive PEth, and response to steroids instead pointed toward ASH.

This case illustrates the role of liver biopsy in resolving diagnostic dilemmas in acute liver injury. Clinicians should be judicious when ordering a liver biopsy, given it is an invasive procedure with potential risks such as hemorrhage (1-2 per 100 biopsies), pneumothorax (0.1-0.3%), and peritonitis (0.1%) [11,12]. However, in cases where the etiology of liver injury remains uncertain, biopsy can help distinguish ASH from autoimmune hepatitis, Wilson’s disease, malignancy, or infiltrative disorders [13]. In our patient, declining synthetic liver function over the hospital course with an unclear etiology provided the impetus for a liver biopsy, and the diagnostic benefits of the procedure outweighed the risks.

Treatment options for ASH, beyond abstinence from alcohol, include corticosteroids, nutritional support, and, less commonly, N-acetylcysteine [14]. Corticosteroids, typically prednisolone, play a crucial role in the short-term management of severe ASH (MELD score >20 or Maddrey’s Discriminant Function >32), reducing mortality [15] by countering hepatic inflammation. However, there are certain scenarios in which corticosteroids may not be appropriate, such as patients with an active infection or kidney injury [14,16]. Response to corticosteroids is assessed at days four and seven of treatment through calculation of the Lille score, with a score less than 0.45 suggestive of adequate response; meanwhile, a Lille score greater than or equal to 0.45 indicates lack of response and potential need for liver transplant [14]. The score incorporates a patient’s age, albumin, change in total bilirubin, creatinine, and prothrombin time [14]. Our patient had a Lille score of 0.012 on day four, indicating a favorable response to prednisolone, and completed a full, 28-day prednisolone course with ultimate improvement in her symptoms and AST, ALT, and ALP.

## Conclusions

Acute liver injury requires a comprehensive evaluation to determine the underlying diagnosis. In this case, the differential diagnosis was pared down to include ASH and Wilson’s disease. Despite abnormal markers for Wilson’s disease, liver biopsy confirmed the diagnosis of ASH. This case highlights the role of liver biopsy in resolving diagnostic uncertainty and guiding management.
